# Biphasic Functional Regulation in Hippocampus of Rat with Chronic Cerebral Hypoperfusion Induced by Permanent Occlusion of Bilateral Common Carotid Artery

**DOI:** 10.1371/journal.pone.0070093

**Published:** 2013-07-30

**Authors:** Jihye Bang, Won Kyung Jeon, In Sun Lee, Jung-Soo Han, Bu-Yeo Kim

**Affiliations:** 1 Herbal Medicine Research Division, Korea Institute of Oriental Medicine, Daejeon, Republic of Korea; 2 Department of Biological sciences, Konkuk University, Seoul, Republic of Korea; Oregon Health & Science University, United States of America

## Abstract

**Background:**

Chronic cerebral hypoperfusion induced by permanent occlusion of the bilateral common carotid artery (BCCAO) in rats has been commonly used for the study of Alzheimer’s disease and vascular dementia. Despite the apparent cognitive dysfunction in rats with BCCAO, the molecular markers or pathways involved in the pathological alternation have not been clearly identified.

**Methods:**

Temporal changes (sham, 21, 35, 45, 55 and 70 days) in gene expression in the hippocampus of rats after BCCAO were measured using time-course microarray analysis. Gene Ontology (GO) and pathway analyses were performed to identify the functional involvement of temporally regulated genes in BCCAO.

**Results:**

Two major gene expression patterns were observed in the hippocampus of rats after BCCAO. One pattern, which was composed of 341 early up-regulated genes after the surgical procedure, was dominantly involved in immune-related biological functions (false discovery rate [FDR]<0.01). Another pattern composed of 182 temporally delayed down-regulated genes was involved in sensory perception such as olfactory and cognition functions (FDR<0.01). In addition to the two gene expression patterns, the temporal change of GO and the pathway activities using all differentially expressed genes also confirmed that an immune response was the main early change, whereas sensory functions were delayed responses. Moreover, we identified *FADD* and *SOCS3* as possible core genes in the sensory function loss process using text-based mining and interaction network analysis.

**Conclusions:**

The biphasic regulatory mechanism first reported here could provide molecular evidence of BCCAO-induced impaired memory in rats as well as mechanism of the development of vascular dementia.

## Introduction

Cognitive impairment is a key feature of dementia, of which Alzheimer’s disease and vascular disease are the two most common forms [1]. Compared with Alzheimer’s disease, the developmental process of vascular dementia has been less well characterized. Vascular dementia has been known to occur when the blood supply to the brain is reduced or inhibited by an impaired vascular system [2]. By mimicking such a pathological condition, various animal models have been developed to explore the underlying mechanism of cognitive impairment in vascular dementia. Permanent occlusion of the bilateral common carotid artery (BCCAO) is a well-established method in rats that is used to investigate the effect of chronic cerebral hypoperfusion on cognitive dysfunction with significant injury to the white matter and hippocampal neuronal damage [3]. Thus, a rat model with chronic cerebral hypoperfusion caused by BCCAO has been widely used for the study of vascular dementia, aging and Alzheimer’s disease as well as for the screening of drugs with therapeutic potential against these neurodegenerative diseases [4], [5].

A potential mechanism for neurodegeneration caused by chronic cerebral hypoperfusion has been proposed in which reduced blood flow can cause neuronal energy failure and promote the production of reactive oxygen species and proinflammatory cytokines by activated microglial cells that, in turn, damage the neuronal cells [3], [7], [8]. Despite this pathological evidence, the molecular factors explaining cognitive dysfunction and pathological alteration by BCCAO in rats has not been clearly identified.

Our previous report indicated that rats with chronic cerebral hypoperfusion induced by BCCAO show impaired spatial memory with activated microglial cells in the white matter including the fimbria of the hippocampus. We also showed the increased expression of choline acetyltransferase in the basal forebrain and activation of mitogen-activated protein kinase (MAPK) in the hippocampus of rats with BCCAO [9]. Other studies have demonstrated that chronic cerebral hypoperfusion can damage a wide range of neuronal regions including the hippocampus, cerebral cortex, white matter area and visual system [6], [10], [11]. However, considering that the hippocampus is highly implicated in spatial learning and memory, and is one of the brain regions that are most sensitive to ischemia, the hippocampus is thought to be the main target brain region of BCCAO-induced damage [3].

Therefore, in the present study, we examined the temporal genetic alterations in the hippocampus of rats with chronic cerebral hypoperfusion induced by BCCAO. Our results revealed a temporally biphasic regulatory pattern of gene expression in the hippocampus. The immune system was activated early in the process; thereafter, the sensory and cognition systems were down-regulated. This novel finding could explain the pathological features induced by chronic cerebral hypoperfusion in rats and provide molecular evidence of the mechanism of vascular dementia.

## Materials and Methods

### Subjects

Twelve-week-old male Wistar rats from OrientBio Inc. (Korea) were used in this study. The rats were housed in a temperature-controlled room at 24±1°C with a relative humidity of 50±10% and a 12 hours dark/light cycle. Food and water were provided *ad libitum* throughout the experiment. This study was performed in strict accordance with the recommendations in the Guide for the Care and Use of Laboratory Animals at the Korea Institute of Oriental Medicine. All experimental procedures were examined and approved by the Institutional Animal Care and Use Committee of the Korea Institute of Oriental Medicine (permit number: KIOM 12-024).

### Surgical Procedure and Brain Preparation

Male Wistar rats were randomly divided into 6 groups (sham, 21, 35, 45, 55 and 70 days after BCCAO) of 12–15 rats each. BCCAO was induced as described previously with some modifications [3], [9], [12]. Briefly, the rats were anesthetized using 5% isoflurane and the bilateral common carotid arteries were tightly double ligated with silk sutures. All efforts were made to minimize pain during surgery. For the control sham group, the same procedure was performed without BCCAO.

### Iba-1 Immunohistochemistry

The brain was post-fixed in 4% paraformaldehyde for 2 days, cryoprotected in phosphate-buffered saline containing 30% sucrose (7 days) at 4°C, and stored at −70°C. Cryosections of the brain (40 µm) were incubated with Iba-1 antibody (Wako, Japan) for 12 hours at 4°C, and incubated with goat anti-rabbit antibody (Cell Signaling Technology, USA). The stained sections were examined under light microscopy (Bx 51; Olympus, Japan) and the number of Iba-1 positive microglial cells was assessed in regions (0.03 mm^2^) of the hippocampus (CA1, CA3) and the dentate gyrus (DG). The difference in the number of Iba-1 positive cells between the experiment group and the sham group was measured using Student’s *t*-test with SPSS 12.0 K software.

### Western Blot Analysis

The hippocampal tissues were homogenated in ice-cold RIPA buffer (Thermo Scientific, USA) and the protein extracts were separated by sodium dodecyl sulfate–polyacrylamide gel electrophoresis and electrically transferred onto polyvinylidene difluoride membranes (Millipore, USA). The membranes were blocked with non-fat dried milk and incubated at 4°C overnight with antibodies specific for inflammatory cytokines (Cell Signaling, USA). The band intensities were detected by using enhanced chemiluminescence reagents (Thermo Scientific, USA).

### Microarray Experiment

Total RNA from the hippocampus from the sham group and each time point group (21, 35, 45, 55 and 70 days) after BCCAO were isolated with TriPure Isolation Reagent in accordance with the manufacturer’s instructions (Roche Applied Science, USA). RNA isolated from 5 rats in each experimental group was pooled prior to microarray analysis to eliminate individual variability. The pooled RNA was amplified and labeled using a Low RNA Input Linear Amplification Kit PLUS (Agilent Technologies, USA) and then hybridized to a microarray (Agilent Rat Whole Genome 44 K; Agilent Technologies, USA) containing approximately 44,000 probes (∼26,600 unique genes), in accordance with the manufacturer’s instructions. The arrays were scanned with an Agilent DNA Microarray Scanner (Agilent Technologies, USA). The dataset is available online at Gene Expression Omnibus (http://www.ncbi.nlm.nih.gov/geo, ID GSE44289).

### Normalization and Clustering of Microarray Data

The raw signal intensities were obtained using Feature Extraction Software (Agilent Technologies, USA) and then quantile normalized [13]. Only array elements >1.4-fold of the local background were considered well measured. Duplicated spots were averaged. The expressional levels of the genes from the experimental groups were compared with those of the sham group and the gene ratios were hierarchically clustered using the CLUSTER, program and visualized using the TreeView program (http://www.eisenlab.org/).

### Time Series Analysis

The short time-series expression miner (STEM) program, which is designed to analyze microarray data from short time-course experiments, was used to identify the temporal patterns composed of similarly expressed genes [14]. The statistical significance of the temporal patterns is calculated using a permutation test (n = 1000) corrected by the false discovery rate (FDR) [14].

### Gene Ontology (GO) Analysis

Identification of enriched GO terms for subgroups of genes was performed using the Functional Annotation Tool of the Database for Annotation, Visualization, and Integrated Discovery (DAVID) program [15] in which a modified Fisher’s exact *p*-*value* and FDR based on the Benjamini-Hochberg procedure were calculated to determine the enrichment of the annotation terms. After a representative subset of the GO terms from the resultant enriched GO terms was identified, the network structure of the non-redundant GO terms subsets was visualized by measuring the semantic similarity in the REVIGO program [16]. Temporal changes of the enriched GO terms were measured by the High-Throughput GoMner algorithm, which uses both the list of genes and the expression ratios to integrate the genes and GO terms. Statistical adjustment is performed to account for testing multiple GO terms from multiple-microarray experiments based on the random sampling of 1,000 iterations [17]. The resultant significant GO terms with a FDR<0.01 were clustered and visualized.

### Pathway Analysis

As with the GO analysis, the enriched pathways for a group of genes were estimated by the same DAVID program in which significantly enriched pathways were identified based on Fisher’s exact test from an input list of genes and statistically adjusted using FDR [15]. Another pathway analysis was conducted using Signaling Pathway Impact Analysis (SPIA) [18]. For a subgroup of genes showing differential expressions, SPIA calculated two statistical measurements (*P_NDE_* and *P_PERT_*) by considering pathway topology with a random bootstrap iteration number of 3,000. *P_NDE_* measures the over-representation of input genes in a specific pathway, while *P_PERT_* measures the abnormal perturbation of a pathway. The global pathway significance *p*-*value*, *P_G_*, was calculated by combining the enrichment and perturbation *p-values*, namely, *P_NDE_* and *P_PERT_*.

We utilized only differentially expressed genes to identify enriched pathways from DAVID or SPIA. However, because the accumulation of small changes by many genes in a pathway could induce significant changes as a whole, we linearly combined the logarithmic expression value of all genes in each pathway and then normalized this value by dividing the number of genes in a pathway, which yielded pathway activity. For genes acting as repressors in a pathway, the weight of −1 was multiplied on the expression values of those genes. To estimate the statistical significance, the permutation-based approach was used, in which the gene labels were randomly permuted 1,000 times [19]. For each permutation, the random pathway activities were estimated. Finally, the FDR of a pathway’s activity was determined by comparing the original activity value with the randomly permutated-values. Pathways with FDR<0.01 were selected and then hierarchically clustered based on pathway activity similarity. The pathway information used in the present study was obtained from the Kyoto Encyclopedia of Genes and Genomes (KEGG, http://www.genome.jp/kegg/).

### Interaction Network and Multiplex Literature Mining

The interaction network structure from a group of genes was constructed using the Cytoscape program [20] with the database maintained by BioGrid (http://www.thebiogrid.org/). Rat gene symbols were converted to human gene symbols to fully utilize the interaction information using the gene orthology database maintained by the Jackson Laboratory (http://www.informatics.jax.org). Input elements and its first interaction neighbors were isolated to form a network structure of the interactions.

To identify the genes associated with neurodegeneration, we used the multiplex literature mining approach. The relationships between gene symbol terms from a group of genes and biological functional terms related to neurodegeneration were measured by the frequency of co-occurrence of these terms in the PubMed by all pairwise comparisons using PubMatix algorithm [21]. The gene terms used in the present study were symbols of genes that were isolated from the expression pattern and appeared in the Title or Abstract of the literature published in 1990–2012. The neurodegeneration-related functional terms that appeared in the Title or Abstract included “dementia”, “Alzheimer”, “Parkinson”, “Huntington”, “Lewy body”, “brain”, “frontotemporal”, “memory”, “cognitive”, “cognition”, “mental”, “behavior”, “dopamine”, “vascular”, “pathology”, “degeneration”, “acetylcholine”, “neurotransmitter”, “acetylcholinesterase”, “neuro”, “neuroscience”, “psychology”, “psychiatry”, “geriatrics” and “gerontology”.

## Results

### Histopathological Examination of the Hippocampus from BCCAO Rats

**Figure 1:**
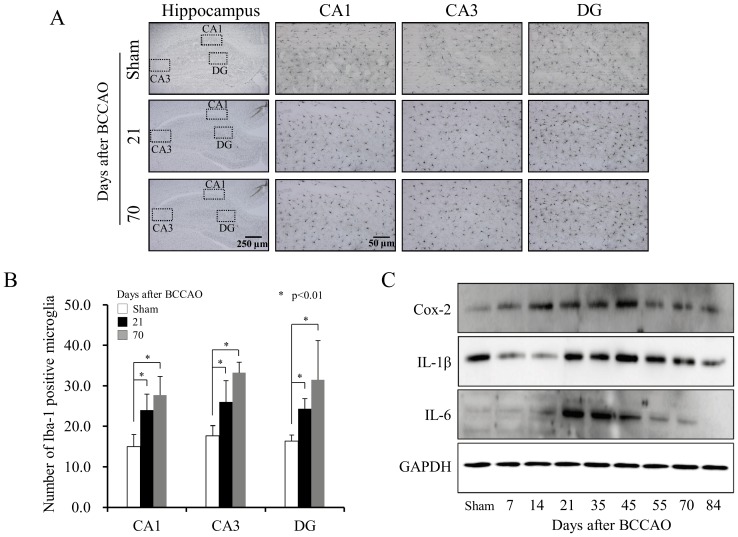
Altered expression of inflammatory proteins in the hippocampus after occlusion of the bilateral common carotid artery (BCCAO). (A) The hippocampus was immunostained with Iba-1 at 21 and 70 days after BCCAO in rats and (B) the number of Iba-1 positive microglia was quantified in each region (0.03 mm^2^) of the hippocampus (CA1, CA3) and the dentate gyrus (DG). The values shown are the mean ± SD of groups of 8 rats. The groups were compared using Student’s *t*-test, with *p*-*values*<0.01 being considered significant. (C) The protein expression of cyclooxygenase-2 (Cox-2), interleukin (IL)-1β and IL-6 in the hippocampus was measured in a time-dependent manner in rats with BCCAO.

The histopathological changes of the hippocampus by BCCAO in rats were measured by immunostaining and western blot analysis. The staining pattern of Iba-1, specific for the microglia, indicated increased- and activated-microglia in the hippocampus of rats with BCCAO (Figures 1A and 1B). The temporal expression of inflammatory cytokines also supports immune response activation in which the levels of cyclooxygenase-2 (Cox-2), Interleukin (IL)-1β and IL-6 peaked at 21–45 days after the BCCAO surgical procedure in rats (Figure 1C). From this result, we determined the time points to be used in the experiments as 21, 35, 45, 55 and 70 days after BCCAO.

### Expression Profile of Genes in the Hippocampus from BCCAO Rats

**Figure 2:**
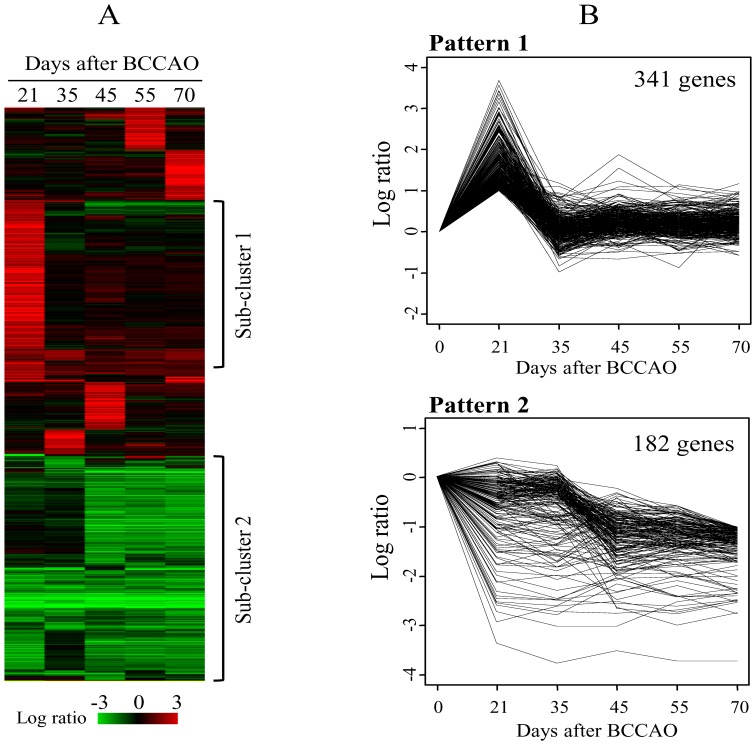
Temporal expression of genes in the hippocampus after BCCAO. (A) A total of 1,583 genes that showed at least 2-fold ratio variation when compared with those of the sham group in at least 1 of 5 samples from the 21, 35, 45, 55 and 70 days time points after BCCAO were hierarchically clustered. Two sub-clusters were indicated as Sub-cluster 1 and Sub-cluster 2. The columns represent individual samples, while the rows represent genes. The red and green colors reflect high and low expression levels, respectively, as indicated by the scale bars. (B) Temporally altered genes were classified using the Short Time-series Expression Miner (STEM) analysis into two patterns (false discovery rate [FDR]<0.001). Pattern 1 was composed of 341 genes and Pattern 2 was composed of 182 genes.

The overall gene expression according to time after BCCAO is shown in Figure 2A, in which the two gene sub-clusters were identified. Sub-cluster 1 consisted of genes up-regulated at 21 days that returned to the control level thereafter. Sub-cluster 2 was composed of genes that were down-regulated over all time points or in a time-dependent pattern. For a more quantitative approach, we conducted time series analysis of the gene expression. Consistent with the clustering pattern, the two major gene expression patterns were identified (FDR<0.001). Pattern 1 was composed of 341 genes that were up-regulated at 21 days after the surgical procedure, while Pattern 2 was composed of 182 genes that were down-regulated in a time-dependent manner. Expression plots for the two gene patterns are shown in Figure 2B. The full list of genes contained in the two expression patterns is shown in Table S1.

### GO Analysis

The gene functions in the two expression patterns were measured in terms of GO. Table 1 lists the top 10 statistically significant categories of GO terms (FDR<0.01). (See Table S2 for a full list of enriched GO terms). Pattern 1 was mainly associated with immune-related GO terms including immune response, defense response and inflammatory response. In contrast, Pattern 2 consisted of sensory-related enriched GO terms such as sensory perception, cognition and detection of chemical stimulus in sensory perception.

**Table 1:** Top 10 GO-terms significantly enriched (false discovery rate [FDR]<0.01) in Pattern 1 and Pattern 2 in the hippocampus of rats after BCCAO surgery.

GO ID	GO terms	*p-value* *	FDR**
**Pattern 1**			
GO:0006955	Immune response	1.58E-29	2.71E-26
GO:0006952	Defense response	2.23E-26	3.85E-23
GO:0006954	Inflammatory response	5.66E-21	9.76E-18
GO:0002252	Immune effector process	1.35E-19	2.33E-16
GO:0009611	Response to wounding	1.48E-18	2.55E-15
GO:0002443	Leukocyte mediated immunity	1.13E-15	1.91E-12
GO:0002684	Positive regulation of immune system process	2.67E-15	4.60E-12
GO:0002253	Activation of immune response	1.36E-14	2.35E-11
GO:0050778	Positive regulation of immune response	7.54E-14	1.30E-10
GO:0002449	Lymphocyte mediated immunity	1.10E-13	1.89E-10
**Pattern 2**			
GO:0007186	G-protein coupled receptor protein signaling pathway	9.83E-16	1.55E-12
GO:0007600	Sensory perception	1.03E-12	1.60E-09
GO:0007166	Cell surface receptor linked signal transduction	1.84E-12	2.86E-09
GO:0007606	Sensory perception of chemical stimulus	5.77E-12	8.96E-09
GO:0050890	Cognition	6.95E-12	1.08E-08
GO:0050911	Detection of chemical stimulus involved in sensory perception of smell	3.98E-11	6.17E-08
GO:0050907	Detection of chemical stimulus involved in sensory perception	5.51E-11	8.56E-08
GO:0009593	Detection of chemical stimulus	8.65E-11	1.34E-07
GO:0007608	Sensory perception of smell	1.05E-10	1.62E-07
GO:0051606	Detection of stimulus	1.10E-10	1.70E-07

* *p-values* were calculated using Fischer’s test.

** FDR corrections were calculated using the Benjamini-Hochberg procedure in DAVID program [15].

### Temporal GO Analysis

**Figure 3:**
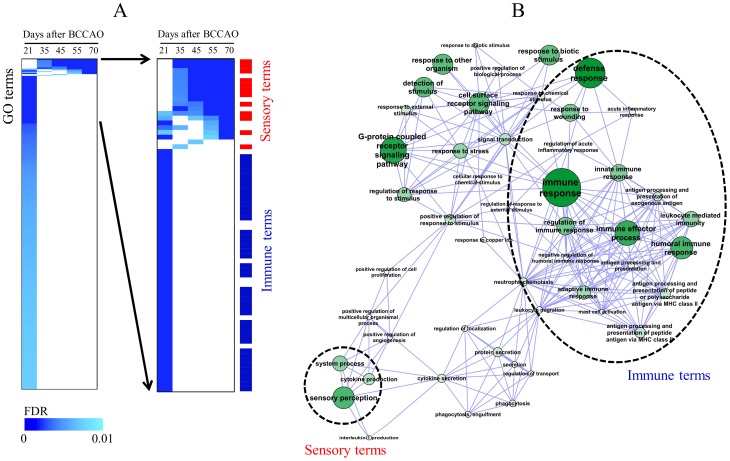
Distribution of altered Gene ontology (GO) terms in the hippocampus after BCCAO. (A) Differentially-expressed genes with a 2-fold increase and decrease at each time point after BCCAO were subjected to analysis using GoMiner. The columns represent individual samples, while the rows represent statistically significant GO categories (FDR<0.01). The statistical significance was represented in color gradient with a scale bar. The positions of the sensory or immune-related terms are indicated in a detailed view. (B) The network structure of non-redundant GO terms was constructed from all enriched GO terms at 21 and 70 days after BCCAO (FDR<0.01) using the REIVGO program. The node size and color thickness were proportional to the statistical significance of each node. The clusters of the sensory terms and immune terms are indicated in dotted circles.

In addition to Patterns 1 and 2, we observed the temporal change of GO terms (FDR<0.01), using all differentially expressed genes according to elapsed time after BCCAO surgery in Figure 3A. Immune functions were clearly enriched at 21 days after BCCAO surgery. Thereafter, the sensory categories were altered in a time-dependent manner. (The full list of GO terms is depicted in Figure S1). However, because these GO categories included redundant terms, we obtained non-redundant GO terms (FDR<0.01) at 21 and 70 days after BCCAO by implementing the REVIGO program and measured the functional relationship of these terms in the network structure (Figure 3B). Interestingly, the sensory perception-associated GO term was interconnected with a large complex cluster of immune-related GO terms, which might implicate the possible connection between the two biological functions.

### Pathways Analysis

We examined the functional changes induced in the hippocampus of rats with BCCAO using pathway analysis. Table 2 lists the enriched pathways (FDR<0.01) in Patterns 1 and 2. Consistent with the GO results, the immune system-related pathways such as the complement pathway (KEGG 04610), systemic lupus erythematosus pathway (KEGG 05322), and natural killer cell pathways (KEGG 04650), were significantly enriched in Pattern 1. The genes involved in sensory system-related pathways such as olfactory transduction pathway (KEGG 04740) and neuroactive ligand-receptor interaction pathway (KEGG 04080), were enriched in Pattern 2.

**Table 2:** Pathway analysis in the hippocampus after BCCAO surgery in a rat model.

KEGG ID	Pathway	*p-value* *	FDR**
**Pattern 1**			
04610	Complement and coagulation cascades	4.60E-11	4.37E-09
05322	Systemic lupus erythematosus	1.96E-08	9.31E-07
04650	Natural killer cell mediated cytotoxicity	6.59E-05	2.08E-03
04062	Chemokine signaling pathway	2.47E-04	5.85E-03
**Pattern 2**			
04740	Olfactory transduction	2.82E-07	2.62E-04
04080	Neuroactive ligand-receptor interaction	1.90E-04	3.38E-03

* *p-values* were calculated using Fischer’s test.

** FDR corrections were calculated using the Benjamini-Hochberg procedure [15].

**Figure 4:**
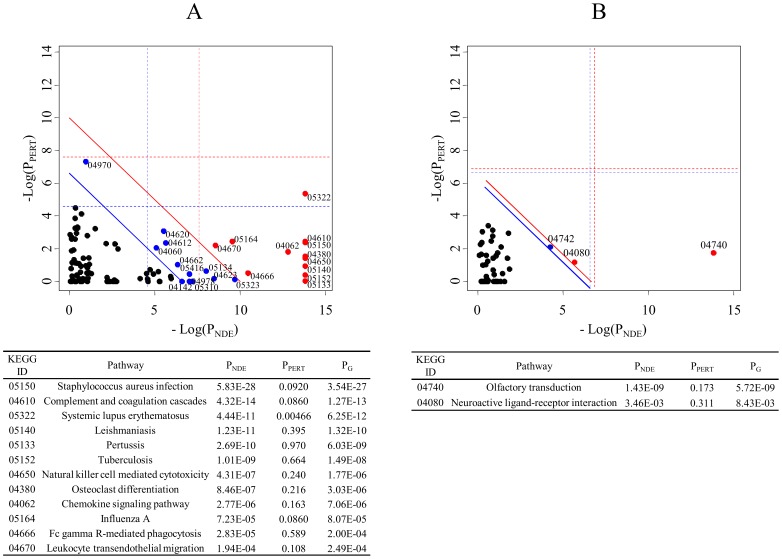
Pathways altered in the hippocampus after BCCAO. Pathways involved in Pattern 1 (A) and Pattern 2 (B) were analyzed via the Signaling Pathway Impact Analysis (SPIA) program. The horizontal axis represents pathway over-representation (*P_NDE_*), while the vertical axis indicates pathway perturbation (*P_PERT_*). The dotted horizontal and vertical lines represent the corrected thresholds (1%) of significance (red color for Bonferroni and blue for FDR correction) for each axis value. The red and blue circles at the right of the oblique lines are significant pathways after the same correction (1%) of the global *p*-*values*, *P_G_* (red line for Bonferroni and blue line for FDR correction). *P_G_*, representing pathway rank, was calculated from the combined probability of both *P_NDE_* and *P_PERT_*. The list of pathways for the red circles is shown below.

For a more systematic analysis of the pathways, we conducted an SPIA pathway analysis, which calculates a significant *p*-*value* of a pathway using its topology. Using genes in Patterns 1 or 2 as input genes, the statistically significant pathways were identified (Figure 4, in which red and blue circles with KEGG ID represent significant pathways after Bonferroni and FDR correction, respectively). In Pattern 1, the immune- or infection-related pathways were significant (*P_G_*<0.01, FDR<0.01), which included the *Staphylococcus aureus* infection pathway (KEGG 05150), complement pathway (KEGG 04610), and systemic lupus erythematosus pathway (KEGG 05322). In Pattern 2, the olfactory transduction pathway (KEGG 04740) and neuroactive ligand-receptor interaction pathway (KEGG 04080) were significant (*P_G_*<0.01, FDR<0.01), consistent with the result of simple pathways enrichment analysis (Table 2). The positions of the individual genes in these statistically significant pathways are depicted in Figure S2.

### Temporal Change of Pathway Activity

**Figure 5:**
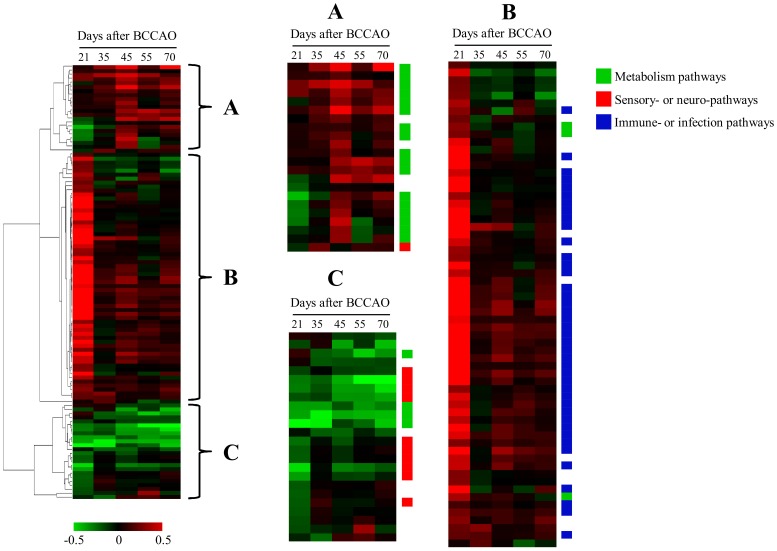
Pathway activities temporally altered in the hippocampus after BCCAO. Pathway activities were calculated by linearly combining gene expression level and then hierarchically clustered. The columns represent individual samples, while the rows represent the pathways. The red and green colors reflect high and low activity levels, respectively, as indicated by the scale bars with arbitrary unit. Three clusters (A, B, and C) are shown in detail. The positions of the pathways were colored green for metabolic pathways, red for sensory- or neuro-related pathways and blue for immune- or infection-related pathways.

The sequential change of pathway activities according to time after BCCAO was measured by linearly combining the expression values of all genes as an activity index of the pathway. With the statistical significance of FDR<0.01, three sub-clusters of pathways were grouped based on the similarity of temporal pathway activities (Figure 5). Sub-cluster A, composed of pathways showing increased activities with time, was mainly associated with the metabolism involving lipoic acid, drug, glutathione, etc. Sub-cluster B consisted of immune- and infection-related pathways culminating at 21 days after BCCAO surgery. Pathways with temporally decreased activities such as the taste transduction, olfactory transduction, phototransduction and neurotransmitter-associated pathways were included in Sub-cluster C. A full list of pathways is shown in Figure S3.

### Network and Text-based Identification of Key Elements

**Figure 6:**
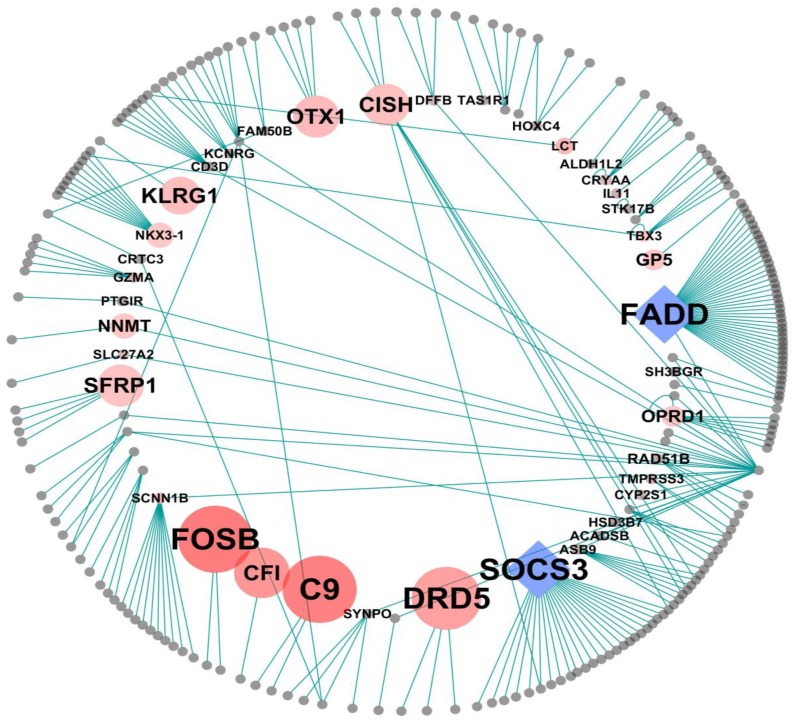
Interaction network of the gene products in Pattern 2. The inner circle was composed of gene products with at least one neighbor from Pattern 2, while the outer circle was composed of first neighbors directly interacting with the inner nodes from the BioGrid database. The size and color thickness of the inner nodes represent the frequency of the co-occurrence of both gene symbols and neurodegeneration-related key terms in the PubMed database. Among these highly referenced nodes, the core nodes with >10 interaction edges, *FADD* and *SOCS3*, are indicated as blue diamonds.

Although we focused on the biological functions within the hippocampus of rats with BCCAO, it is still important to identify the core genes playing key roles in these functional changes. At first, we constructed an interaction network among genes in Pattern 2 and its first neighbors, combined with the results of a text-based literature search (Figure 6). The nodes on the inner circle represent the genes with at least one neighbor from Pattern 2, while the outer circle of nodes represents its first interacting neighbors. The node sizes and colors in the inner circle corresponded to the frequency of co-occurrence of gene symbols and key-terms in the PubMed database. The most commonly used neurodegeneration-related key terms that appeared in the Title of Abstract of the literature were “dementia,” “Alzheimer,” “Parkinson,” “Huntington,” “Lewy body,” “brain,” “frontotemporal,” “memory,” “cognitive,” “cognition,” “mental,” “behavior,” “dopamine,” “vascular,” “pathology,” “degeneration,” “acetylcholine,” “neurotransmitter,” “acetylcholinesterase,” “neuro,” “neuroscience,” “psychology,” “psychiatry,” “geriatrics,” and “gerontology.” It was evident that many of the genes in the inner circle of Pattern 2 have been repeatedly reported to be associated with search terms. For example, *FOSB, CFI, C9, DRD5, FADD, OTX1,* and *SOCS3* were genes repeatedly associated with the functional terms of neurodegeneration in the literatures. Among them, *FADD* and *SOCS3*, the blue diamond in Figure 6, interacts with >10 other gene products and could act as key core elements in sensory and cognition function impaired by BCCAO in rats.

## Discussion

A persistent ischemic condition in the brain can generate reactive oxygen species and lethal neuroinflammation in the neuronal cells, which induces impairment of the memory process and eventually leads to the development of vascular dementia [3], [7], [8]. Chronic cerebral hypoperfusion by BCCAO in rats is a well-established model for the study of vascular dementia. However, the types and time of involvement of molecules in this process remain obscure.

In the present study, expression of IL-1β and IL-6, well-known neuroinflammation markers, culminated at 21–45 days (Figure 1). Also, our previous report showed that memory impairments and neuroinflammation are evident at 12 weeks after BCCAO in rats [9]. Therefore, we tried to measure the temporal changes of gene expression by focusing on that period. From temporal profile of gene expression after BCCAO surgery, the two major patterns could be separated with biological significance. Pattern 1 was associated with the immune response, while Pattern 2 was associated with the sensory and cognition functions (Figure 2, Table 1). Considering that the genes in Patten 1 were up-regulated at an early time, the immune response was considered to be early responsive to BCCAO, while dysfunction of sensory and cognition functions in the hippocampus was delayed after BCCAO in rats as inferred from gene expression of Pattern 2. This delayed-impairment of sensory function might result from the sequential process of neuronal degeneration initiated by destructive inflammation. In addition to the genes included in only Pattern 1 or Pattern 2, using all differentially expressed genes at each time point, we obtained similar functional alterations (Figures 3 and 5). The temporal changes of GO terms demonstrated that sensory perception especially that involved in olfactory function, was enriched proportionally according to the elapsed time after surgical procedure. On the other hand, the immune-associated terms were exclusively enriched at an early time. Interestingly, the network structure of redundancy-removed GO terms suggests a functional connection between immune-responsive clusters and sensory-perception GO terms (Figure 3), implicating a possible biological relevance of these two major functions.

The temporal pattern of pathway activities using all deregulated genes also confirmed the activation of early immune response pathways and delayed de-activation of sensory pathways (Figure 5). However, many immune response pathways were still in the activated condition even later in time, although the strength was reduced. This suggests that immune response functions could be continually involved in pathological changes of the hippocampus in rats with BCCAO and could explain the increased staining of Iba-1 and protein level of IL-1β and IL-6 at delayed time points (Figure 1). On the contrary, activities of sensory pathways including the olfactory transduction, taste transduction and phototransduction pathways were already diminished from early time, and thereafter, more severely de-activated (Figure 5 and Figure S3). This gradual change of molecular functions evidenced the biphasic regulatory mechanism. Interestingly, we also measured impairment of the optic tract in the brain of rats by BCCAO (data not shown). These results could provide molecular evidence of the well-known visual damage induced by BCCAO in rats [4], [10].

In addition to the two major clusters of pathways, another subgroup of pathways (Sub-cluster A in Figure 5) was identified, which was mainly composed of metabolic pathways. Although the role of these pathways on the pathology of the hippocampus was unknown, they demonstrate a significant alteration of many biological functions in the hippocampus by BCCAO. Moreover, considering that other neurodegenerative disease pathways, such as the Alzheimer’s, Huntington’s and Parkinson’s pathways, were not involved in the pathological process by BCCAO, the molecular process of cognitive impairment by BCCAO might be different from that of other neurodegenerative diseases.

In addition to biological function, we identified FADD and SOCS3 as key core elements in the sensory impairment process by BCCAO (Figure 6). FADD was reportedly involved in brain dopamine signaling [22], [23], while SOCS3 can promote progression toward human immunodeficiency virus (HIV)-associated dementia in patients with HIV [24]. Interestingly, CISH, a known suppressor of the SOCS protein family [25], [26], was also included as a core node in Figures 6. In addition to these key core elements, many other core genes from the network structure in Figure 6 have been known to be involved in brain function, sensory impairment and vascular function. For example, the increased expression of KLRG1, which belongs to the killer cell lectin-like receptor family, is commonly considered a senescence marker in patients with Alzheimer’s disease [27]. Complement C9 deposition in the hippocampus contributes to responses to brain injury and Alzheimer’s disease [28] and is induced in human neuronal cells by inflammatory stimuli [29]. Complement factor I (CFI), another protein of the complement systems, is also involved in age-related macular degeneration [30], [31]. Although these core genes like C9 and CFI were from Pattern 2 which was related to sensory function, the immunological roles of these genes imply that immune functions could be critical on cognitive impairment by BCCAO. Another core element, OTX1, acting as a transcription factor, play a key role in brain and sensory organ development [32], [33]. In addition to brain-related function, SFRP1 acts as a potent angiogenic factor on vascularization after ischemic or hypoxia events induced by cerebral hypoperfusion [34], which could evidence the possible involvement of SFRP1 in the process of vascular dementia. However, many genes included in the olfactory transduction pathway (KEGG 04740) and neuroactive ligand-receptor interaction pathway (KEGG 04080), which were enriched in Pattern 2, have not been previously reported to be associated with a neurodegenerative disease. Only DRD5 in the neuroactive ligand-receptor interaction pathway was widely studied in cognitive impairment and Alzheimer’s disease [35], [36].

In conclusion, the findings of the present study indicated the biphasic regulatory pattern induced by chronic cerebral hypoperfusion in the hippocampus of rats with BCCAO. Early response consisted of immune systems activation, while the delayed response consisted of down-regulated sensory systems. Although the role of the immune response on the late onset of sensory impairment should be revealed in greater detail, the present molecular events in the hippocampus could provide clues to understanding the mechanism of the impaired spatial memory by chronic cerebral hypoperfusion in rats with BCCAO.

## Supporting Information

Figure S1 **List of Gene Ontology (GO) terms that were temporally altered in the hippocampus of rats after occlusion of the bilateral common carotid artery (BCCAO) surgery.** GO terms in red and blue represent sensory and immune-related terms, respectively.
(TIF)

Figure S2 **Pathways enriched in Patterns 1 and 2.** The position of each gene is colored green in the pathways.
(TIF)

Figure S3 **Full list of pathways temporally altered in the hippocampus after BCCAO surgery.** The names of the metabolic pathways are colored green, those of the sensory- or neuro-related pathways are colored red and those of the immune- or infection-related pathways are colored blue.
(TIF)

Table S1 **List of genes in Pattern 1 and Pattern 2 in the hippocampus of rats with BCCAO surgery.**
(DOCX)

Table S2 **Full list of GO-terms significantly enriched (FDR<0.01) in Pattern 1 and Pattern 2 in the hippocampus of rats with BCCAO surgery.**
(DOCX)
