# Supplementary figures and images for: Biphasic Functional Regulation in Hippocampus of Rat with Chronic Cerebral Hypoperfusion Induced by Permanent Occlusion of Bilateral Common Carotid Artery
Source: PLoS One. 2013 Jul 30;8(7):e70093. doi: 10.1371/journal.pone.0070093 (PMC3728362; doi:10.1371/journal.pone.0070093)

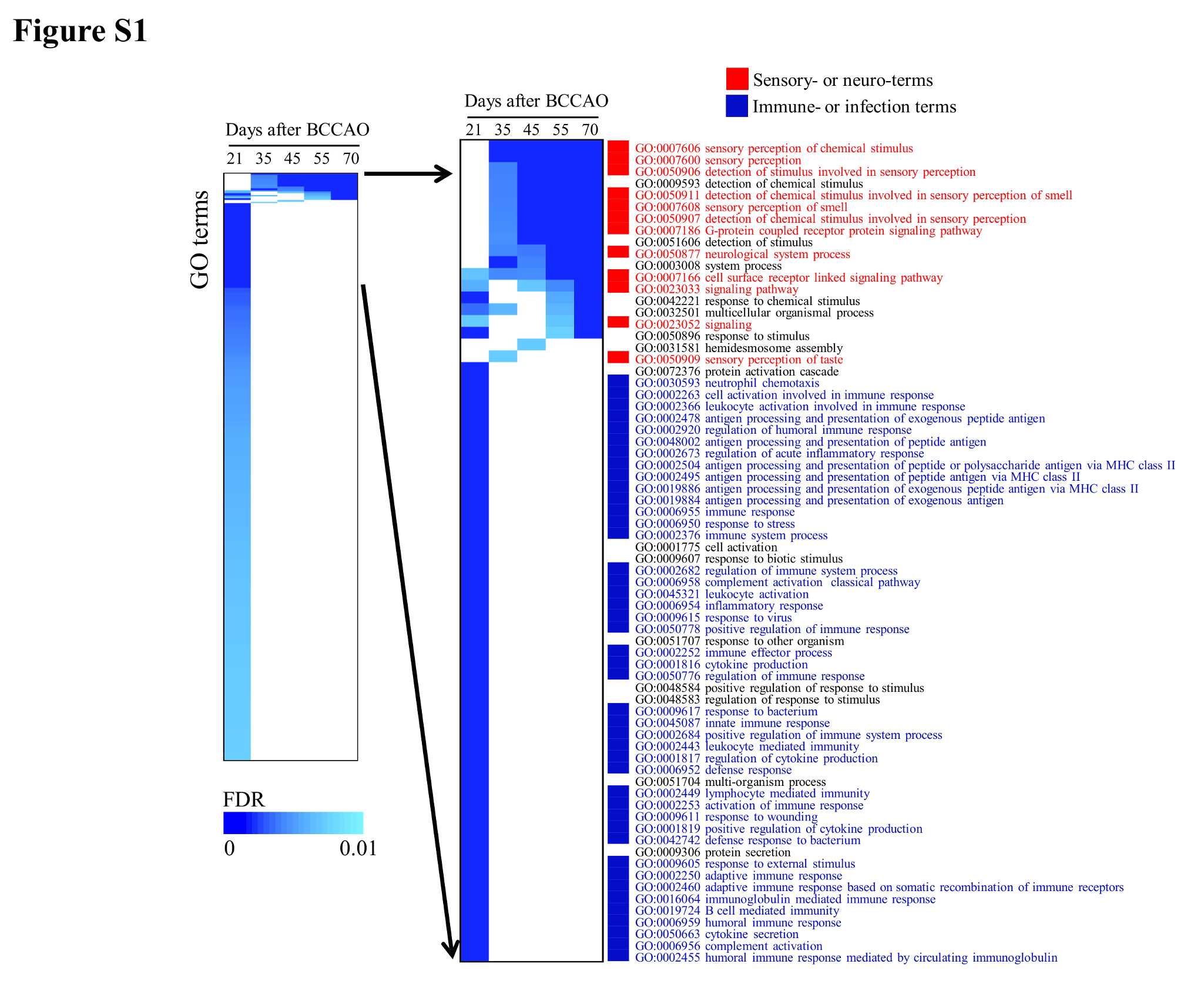

Supplement: Figure S1 — List of Gene Ontology (GO) terms that were temporally altered in the hippocampus of rats after occlusion of the bilateral common carotid artery (BCCAO) surgery. GO terms in red and blue represent sensory and immune-related terms, respectively. (TIF) [file pone.0070093.s001.tif]

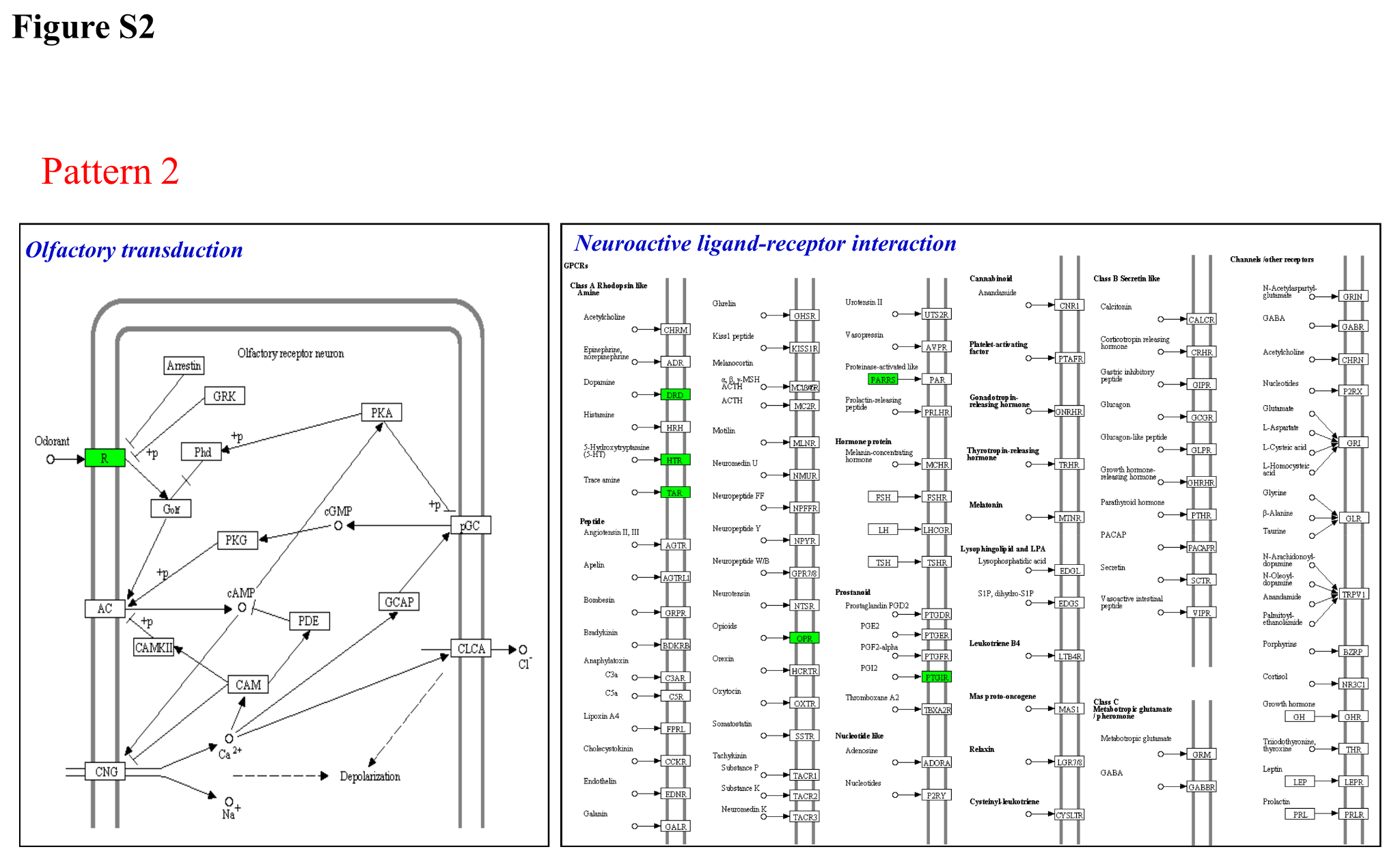

Supplement: Figure S2 — Pathways enriched in Patterns 1 and 2. The position of each gene is colored green in the pathways. (TIF) [file pone.0070093.s002.tif]

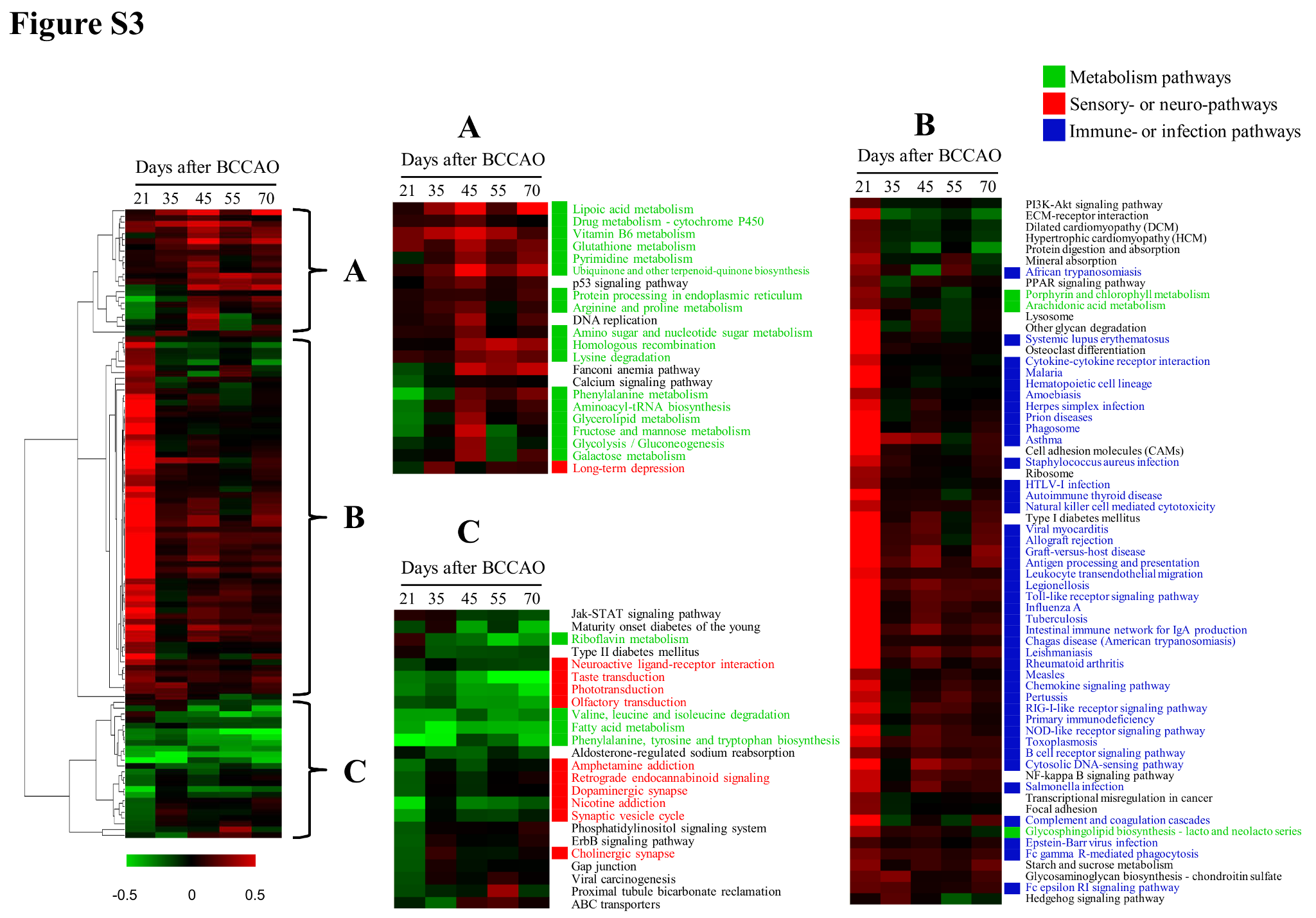

Supplement: Figure S3 — Full list of pathways temporally altered in the hippocampus after BCCAO surgery. The names of the metabolic pathways are colored green, those of the sensory- or neuro-related pathways are colored red and those of the immune- or infection-related pathways are colored blue. (TIF) [file pone.0070093.s003.tif]
