# Supplementary material for: Biphasic Functional Regulation in Hippocampus of Rat with Chronic Cerebral Hypoperfusion Induced by Permanent Occlusion of Bilateral Common Carotid Artery
Source: PLoS One. 2013 Jul 30;8(7):e70093. doi: 10.1371/journal.pone.0070093 (PMC3728362; doi:10.1371/journal.pone.0070093)
Supplement: Table S1 — List of genes in Pattern 1 and Pattern 2 in the hippocampus of rats with BCCAO surgery. (DOCX) [file pone.0070093.s004.docx]

Table S1. List of genes in Pattern 1 and Pattern 2 in the hippocampus of rats with BCCAO surgery.

| **Pattern** | **Symbol** | **Accession** | **Entrez ID** |
| --- | --- | --- | --- |
| 1 | A2m | NM_012488 | 24153 |
| 1 | Abcg3l2 | NM_001014133 | 360910 |
| 1 | Adam33 | NM_001107776 | 311425 |
| 1 | Adm | NM_012715 | 25026 |
| 1 | Aebp1 | NM_001100970 | 305494 |
| 1 | Aif1 | NM_017196 | 29427 |
| 1 | Aldh1a2 | NM_053896 | 116676 |
| 1 | Anxa1 | NM_012904 | 25380 |
| 1 | Anxa2 | NM_019905 | 56611 |
| 1 | Anxa3 | NM_012823 | 25291 |
| 1 | Apbb1ip | NM_001100577 | 307171 |
| 1 | Apobec1 | NM_012907 | 25383 |
| 1 | Apol9a | NM_001025066 | 503164 |
| 1 | Arhgdib | NM_001009600 | 362456 |
| 1 | Arl11 | NM_001013433 | 364396 |
| 1 | Aspg | NM_144750 | 246266 |
| 1 | Atf3 | NM_012912 | 25389 |
| 1 | B3gnt5 | NM_053932 | 116740 |
| 1 | Bcl3 | NM_001109422 | 680611 |
| 1 | Blnk | NM_001025767 | 499356 |
| 1 | Bst2 | NM_198134 | 378947 |
| 1 | C1qa | NM_001008515 | 298566 |
| 1 | C1qb | NM_019262 | 29687 |
| 1 | C1qc | NM_001008524 | 362634 |
| 1 | C1r | NM_001134555 | 312705 |
| 1 | C1s | NM_138900 | 192262 |
| 1 | C3 | NM_016994 | 24232 |
| 1 | C3ar1 | NM_032060 | 84007 |
| 1 | C4b | NM_031504 | 24233 |
| 1 | C5ar1 | NM_053619 | 113959 |
| 1 | C6 | NM_176074 | 24237 |
| 1 | C7 | XM_001054007 | 117517 |
| 1 | Capg | NM_001013086 | 297339 |
| 1 | Casp4 | NM_053736 | 114555 |
| 1 | Ccdc81 | NM_001014021 | 308810 |
| 1 | Ccl19 | NM_001108661 | 362506 |
| **Pattern** | **Symbol** | **Accession** | **Entrez ID** |
| 1 | Ccl2 | NM_031530 | 24770 |
| 1 | Ccl3 | NM_013025 | 25542 |
| 1 | Ccl4 | NM_053858 | 116637 |
| 1 | Ccl7 | NM_001007612 | 287561 |
| 1 | Ccr5 | NM_053960 | 117029 |
| 1 | Ccr6 | NM_001013145 | 308163 |
| 1 | Cd14 | NM_021744 | 60350 |
| 1 | Cd37 | NM_017124 | 29185 |
| 1 | Cd53 | NM_012523 | 24251 |
| 1 | Cd74 | NM_013069 | 25599 |
| 1 | Cd84 | NM_001192006 | 501872 |
| 1 | cDNA clone | BG378999 |  |
| 1 | cDNA clone | BM387339 |  |
| 1 | Cfb | NM_212466 | 294257 |
| 1 | Cfd | NM_001077642 | 54249 |
| 1 | Cfh | NM_130409 | 155012 |
| 1 | Ch25h | NM_001025415 | 309527 |
| 1 | Cidea | NM_001170467 | 291541 |
| 1 | Cklf | NM_139111 | 245978 |
| 1 | Clec12a | NM_001134716 | 680338 |
| 1 | Clec4a3 | NM_001005891 | 362431 |
| 1 | Clec7a | NM_001173386 | 502902 |
| 1 | Cmtm7 | NM_001109300 | 501065 |
| 1 | Cnbd1 | XM_001070898 | 689462 |
| 1 | Cndp1 | NM_001007687 | 307212 |
| 1 | Col15a1 | XM_001066530 | 298069 |
| 1 | Col8a1 | NM_001107100 | 304021 |
| 1 | Cp | NM_012532 | 24268 |
| 1 | Cpsf4l | XM_001081632 | 287799 |
| 1 | Cpz | NM_031766 | 83575 |
| 1 | Cryab | NM_012935 | 25420 |
| 1 | Ctsc | NM_017097 | 25423 |
| 1 | Ctsd | NM_134334 | 171293 |
| 1 | Ctse | NM_012938 | 25424 |
| 1 | Ctss | NM_017320 | 50654 |
| 1 | Ctsz | NM_183330 | 252929 |
| 1 | Cxcl16 | NM_001017478 | 497942 |
| 1 | Cxcl17 | NM_001107491 | 308436 |
| 1 | Cyba | NM_024160 | 79129 |
| 1 | Cybrd1 | NM_001011954 | 295669 |
| **Pattern** | **Symbol** | **Accession** | **Entrez ID** |
| 1 | Cyp2j10 | NM_001134980 | 313373 |
| 1 | Dapp1 | NM_001108568 | 362046 |
| 1 | Defb21 | NM_001037512 | 641636 |
| 1 | Dennd2d | NM_001107714 | 310772 |
| 1 | Derl2 | XM_001079920 | 691956 |
| 1 | Dnajc22 | NM_001014204 | 362998 |
| 1 | Dnase2a | NM_138539 | 171575 |
| 1 | Dock8 | NM_001037793 | 499337 |
| 1 | Dpep2 | NM_001011928 | 291984 |
| 1 | Emp3 | NM_030847 | 81505 |
| 1 | Endou | XM_001056619 | 680317 |
| 1 | ENSRNOT00000002917 | XM_001056810 |  |
| 1 | ENSRNOT00000003948 | EF094477 |  |
| 1 | ENSRNOT00000005511 | XM_234253 |  |
| 1 | ENSRNOT00000037316 | XM_223483 |  |
| 1 | ENSRNOT00000037518 | XM_001062561 |  |
| 1 | ENSRNOT00000039335 | XM_002724498 |  |
| 1 | ENSRNOT00000046884 | XM_237757 |  |
| 1 | ENSRNOT00000047793 | XR_086196 |  |
| 1 | ENSRNOT00000049859 | XM_002724710 |  |
| 1 | ENSRNOT00000051382 | XM_001073551 |  |
| 1 | ENSRNOT00000059330 | XM_001061048 |  |
| 1 | ENSRNOT00000059648 | XM_231714 |  |
| 1 | Epsti1 | NM_001044257 | 498547 |
| 1 | F10 | NM_017143 | 29243 |
| 1 | Fam46a | NM_001106844 | 300870 |
| 1 | Fcer1g | NM_001131001 | 25441 |
| 1 | Fcgr2a | XM_002724851 | 116591 |
| 1 | Fcgr2b | NM_175756 | 289211 |
| 1 | Fcgr3a | NM_207603 | 304966 |
| 1 | Fes | NM_001108488 | 361597 |
| 1 | Fgd2 | NM_001107617 | 309653 |
| 1 | Fhod1 | NM_001191600 | 291964 |
| 1 | Fmod | NM_080698 | 64507 |
| 1 | Folr1 | NM_133527 | 171049 |
| 1 | Foxj1 | NM_053832 | 116557 |
| 1 | Fut7 | NM_199491 | 296564 |
| 1 | Fxyd1 | NM_031648 | 58971 |
| 1 | Fxyd3 | NM_172317 | 116831 |
| 1 | Fxyd5 | NM_021909 | 60338 |
| **Pattern** | **Symbol** | **Accession** | **Entrez ID** |
| 1 | Fyb | NM_001109176 | 499537 |
| 1 | Gadd45a | NM_024127 | 25112 |
| 1 | Gata1 | NM_012764 | 25172 |
| 1 | Gba3 | NM_001106010 | 289687 |
| 1 | Gbp2 | NM_133624 | 171164 |
| 1 | Gmfg | NM_181091 | 113940 |
| 1 | Gnat2 | NM_001108950 | 365901 |
| 1 | Gngt2 | NM_001135767 | 690825 |
| 1 | Gpd1 | NM_022215 | 60666 |
| 1 | Gpnmb | NM_133298 | 113955 |
| 1 | Gpr18 | NM_001079710 | 679957 |
| 1 | Gpr183 | NM_001109386 | 679975 |
| 1 | Gpr84 | NM_001109509 | 688730 |
| 1 | Gpr87 | NM_001107677 | 310443 |
| 1 | Grap | NM_001025749 | 363616 |
| 1 | Grn | NM_017113 | 29143 |
| 1 | Hck | NM_013185 | 25734 |
| 1 | Hcst | NM_001005900 | 474146 |
| 1 | Hlx | NM_001077674 | 364069 |
| 1 | Hmox1 | NM_012580 | 24451 |
| 1 | Hpgds | NM_031644 | 58962 |
| 1 | Hsd3b6 | NM_017265 | 29632 |
| 1 | Hspb1 | NM_031970 | 24471 |
| 1 | Icam1 | NM_012967 | 25464 |
| 1 | Ifi27l2b | NM_206846 | 299269 |
| 1 | Ifi30 | NM_001030026 | 290644 |
| 1 | Ifitm3 | NM_001136124 | 361673 |
| 1 | Ifitm7 | XM_001054983 | 288244 |
| 1 | Igf1 | NM_001082479 | 24482 |
| 1 | Igf2 | NM_031511 | 24483 |
| 1 | Igfbp2 | NM_013122 | 25662 |
| 1 | Igsf10 | NM_198768 | 310448 |
| 1 | Il18 | NM_019165 | 29197 |
| 1 | Il1rn | NM_022194 | 60582 |
| 1 | Irf5 | NM_001106586 | 296953 |
| 1 | Irf7 | NM_001033691 | 293624 |
| 1 | Irf8 | NM_001008722 | 292060 |
| 1 | Isg15 | NM_001106700 | 298693 |
| 1 | Itgam | ENSRNOT00000026748 | 25021 |
| 1 | Itgb2 | NM_001037780 | 309684 |
| **Pattern** | **Symbol** | **Accession** | **Entrez ID** |
| 1 | Kcnj5 | NM_017297 | 29713 |
| 1 | Klra2 | NM_001009486 | 494194 |
| 1 | Krt77 | NM_001008807 | 406226 |
| 1 | Laptm5 | NM_053538 | 89783 |
| 1 | Lat2 | NM_173840 | 317676 |
| 1 | Lcat | NM_017024 | 24530 |
| 1 | Lcp1 | NM_001012044 | 306071 |
| 1 | Lcp2 | NM_130421 | 155918 |
| 1 | Lgals1 | NM_019904 | 56646 |
| 1 | Lgals3bp | NM_139096 | 245955 |
| 1 | Lhx5 | NM_139036 | 124451 |
| 1 | LOC363894 | XM_002727356 | 363894 |
| 1 | LOC367975 | NM_001079896 | 367975 |
| 1 | LOC498276 | NM_001135992 | 498276 |
| 1 | LOC498350 | NM_001017498 | 498350 |
| 1 | LOC681186 | XM_001060674 | 681186 |
| 1 | LOC685067 | XR_006095 | 685067 |
| 1 | LOC685157 | NM_001115044 | 685157 |
| 1 | LOC685262 | XM_001063066 | 685262 |
| 1 | LOC685722 | NM_001109479 | 685722 |
| 1 | LOC685819 | XM_001065393 | 685819 |
| 1 | LOC687856 | XM_001080365 | 687856 |
| 1 | LOC690523 | XM_001074680 | 690523 |
| 1 | LOC691107 | XM_001074988 | 691107 |
| 1 | Loxl2 | NM_001106047 | 290350 |
| 1 | Lrg1 | NM_001009717 | 367455 |
| 1 | Lrrc15 | NM_145083 | 246296 |
| 1 | Ly86 | NM_001106128 | 291359 |
| 1 | Lyl1 | NM_001007677 | 304663 |
| 1 | Lyz2 | NM_012771 | 25211 |
| 1 | Mafb | NM_019316 | 54264 |
| 1 | Mall | NM_001014182 | 362211 |
| 1 | Man2b2 | NM_001134971 | 360955 |
| 1 | Mefv | NM_031634 | 58923 |
| 1 | MGC105649 | NM_001008518 | 302884 |
| 1 | MGC108823 | NM_001012353 | 307414 |
| 1 | Mmp14 | NM_031056 | 81707 |
| 1 | Mmp7 | NM_012864 | 25335 |
| 1 | Mpeg1 | NM_022617 | 64552 |
| 1 | Mrgprb5 | NM_001002284 | 404644 |
| **Pattern** | **Symbol** | **Accession** | **Entrez ID** |
| 1 | Mrgprx3 | NM_145787 | 252960 |
| 1 | Ms4a12 | NM_001106337 | 293741 |
| 1 | Ms4a6a | XM_001075462 | 293750 |
| 1 | Ms4a6b | NM_001006975 | 293749 |
| 1 | Ms4a6c | XM_001078213 | 690930 |
| 1 | Mt1a | NM_138826 | 24567 |
| 1 | Mt2A | NM_001137564 | 689415 |
| 1 | Mx1 | NM_173096 | 24575 |
| 1 | Mybpc2 | NM_001106257 | 292879 |
| 1 | Myo1f | NM_001108076 | 314654 |
| 1 | Myo1g | NM_001134843 | 289785 |
| 1 | Ncf1 | NM_053734 | 114553 |
| 1 | Ncf2 | NM_001100984 | 364018 |
| 1 | Nckap1l | NM_001108119 | 315348 |
| 1 | Nlrc4 | NM_001106707 | 298784 |
| 1 | Npc2 | NM_173118 | 286898 |
| 1 | Nr0b2 | NM_057133 | 117274 |
| 1 | Nthl1 | NM_001105728 | 29541 |
| 1 | Nuak1 | NM_001106774 | 299694 |
| 1 | Nupr1 | NM_053611 | 113900 |
| 1 | Oas1a | NM_138913 | 192281 |
| 1 | Oasl2 | NM_001009682 | 304549 |
| 1 | Ogn | NM_001106103 | 291015 |
| 1 | Olr1 | NM_133306 | 140914 |
| 1 | Olr1083 | NM_001000710 | 404959 |
| 1 | Olr1362 | NM_001006598 | 363546 |
| 1 | Olr1421 | NM_001000780 | 405057 |
| 1 | Olr1542 | NM_001000531 | 363763 |
| 1 | Olr267 | NM_001000228 | 293435 |
| 1 | Olr343 | NM_001000254 | 293788 |
| 1 | Olr477 | NM_001000304 | 295747 |
| 1 | Olr529 | NM_001000674 | 404877 |
| 1 | Omd | NM_031817 | 83717 |
| 1 | P2ry12 | NM_022800 | 64803 |
| 1 | P2ry6 | NM_057124 | 117264 |
| 1 | Pabpc1l2b | XM_228576 | 302405 |
| 1 | Parp9 | NM_001103351 | 303905 |
| 1 | Pcdhga5 | NM_001037137 | 291637 |
| 1 | Pla2g7 | NM_001009353 | 301265 |
| 1 | Plek | NM_001025750 | 364206 |
| **Pattern** | **Symbol** | **Accession** | **Entrez ID** |
| 1 | Ppic | NM_001004215 | 291463 |
| 1 | Ppp1r15b | NM_001107175 | 304799 |
| 1 | Prkcd | NM_133307 | 170538 |
| 1 | Prrx2 | NM_001105739 | 113931 |
| 1 | Ptafr | NM_053321 | 58949 |
| 1 | Ptgr1 | NM_138863 | 192227 |
| 1 | Ptpn6 | NM_053908 | 116689 |
| 1 | Ptpn7 | NM_145683 | 246781 |
| 1 | Ptprc | NM_138507 | 24699 |
| 1 | Pycard | NM_172322 | 282817 |
| 1 | Rab32 | NM_001108902 | 365042 |
| 1 | Rac2 | NM_001008384 | 366957 |
| 1 | Rasal3 | NM_001134562 | 314596 |
| 1 | Rassf4 | NM_001024275 | 362423 |
| 1 | Rcan3 | NM_001012746 | 362627 |
| 1 | Reep4 | NM_001025279 | 306014 |
| 1 | RGD1305645 | XM_343562 | 363225 |
| 1 | RGD1309049 | NM_001013956 | 301306 |
| 1 | RGD1309362 | NM_001024884 | 307415 |
| 1 | RGD1559482 | NM_001168285 | 498022 |
| 1 | RGD1560940 | NM_001109298 | 501060 |
| 1 | RGD1561143 | XM_002724830 | 304382 |
| 1 | RGD1561715_predicted | XR_007960 |  |
| 1 | RGD1562552 | NM_001109122 | 498840 |
| 1 | RGD1562726 | NM_001109052 | 498060 |
| 1 | RGD1563378 | XM_001059106 | 302719 |
| 1 | RGD1563581 | XM_225088 | 502110 |
| 1 | RGD1564325 | XM_001062245 | 306259 |
| 1 | RGD1564482 | NR_036617 | 500568 |
| 1 | RGD1565648 | XM_002729212 | 499911 |
| 1 | RGD1565772 | NM_001134545 | 308341 |
| 1 | RGD1566254 | XM_579948 | 499318 |
| 1 | Rgs1 | NM_019336 | 54289 |
| 1 | Ripk3 | NM_139342 | 246240 |
| 1 | Rnaset2 | NM_001106210 | 292306 |
| 1 | Rnf38 | NM_134467 | 171501 |
| 1 | Rsad2 | NM_138881 | 65190 |
| 1 | RT1-Bb | NM_001004084 | 309622 |
| 1 | RT1-Da | NM_001008847 | 294269 |
| 1 | RT1-DOb | NM_001008846 | 365542 |
| **Pattern** | **Symbol** | **Accession** | **Entrez ID** |
| 1 | Runx1 | NM_017325 | 50662 |
| 1 | S100a13 | NM_001191607 | 295213 |
| 1 | S100a3 | NM_053681 | 114216 |
| 1 | S100a4 | NM_012618 | 24615 |
| 1 | S100a6 | NM_053485 | 85247 |
| 1 | Scn7a | NM_031686 | 64155 |
| 1 | Sdf2l1 | NM_001109433 | 680945 |
| 1 | Serpinb1a | NM_001031642 | 291091 |
| 1 | Serping1 | NM_199093 | 295703 |
| 1 | Sfrs1 | NM_001109552 | 689890 |
| 1 | Sgcg | NM_001006993 | 305941 |
| 1 | Sh2b2 | NM_053669 | 114203 |
| 1 | Siglec1 | NM_001107777 | 311426 |
| 1 | Similar to Tes protein (LOC298327) | XM_233288 |  |
| 1 | Skap2 | NM_130413 | 155183 |
| 1 | Slamf9 | NM_001105971 | 289235 |
| 1 | Slc15a3 | NM_139341 | 246239 |
| 1 | Slc22a18 | NM_001004260 | 309131 |
| 1 | Slc22a6 | NM_017224 | 29509 |
| 1 | Slc2a5 | NM_031741 | 65197 |
| 1 | Slc37a2 | XM_576383 | 500973 |
| 1 | Slc6a13 | NM_133623 | 171163 |
| 1 | Slfn2 | NM_001107031 | 303380 |
| 1 | Slfn3 | NM_053687 | 114247 |
| 1 | Slfn8 | NM_001013970 | 303378 |
| 1 | Sp110 | NM_001034137 | 301570 |
| 1 | Sp140 | NM_001012133 | 316580 |
| 1 | Spint1 | NM_001004265 | 311331 |
| 1 | St14 | NM_053635 | 114093 |
| 1 | Syt9 | NM_053324 | 60564 |
| 1 | Tac4 | NM_172328 | 282829 |
| 1 | Tbxas1 | NM_012687 | 24886 |
| 1 | TC597252 | XM_214813 |  |
| 1 | TC613234 | XM_217035 |  |
| 1 | Tex14 | XM_001081119 | 287603 |
| 1 | Tgfb1 | NM_021578 | 59086 |
| 1 | Tgif1 | NM_001015020 | 316742 |
| 1 | Tifab | NM_001025029 | 364674 |
| 1 | Timp1 | NM_053819 | 116510 |
| 1 | Tlr13 | XM_002727615 | 317227 |
| **Pattern** | **Symbol** | **Accession** | **Entrez ID** |
| 1 | Tlr3 | NM_198791 | 364594 |
| 1 | Tmem156 | NM_001025138 | 498365 |
| 1 | Tmem176a | NM_001039008 | 297077 |
| 1 | Tmem176b | NM_134390 | 171411 |
| 1 | Tmem87a | XM_345422 | 366170 |
| 1 | Tnfaip2 | NM_001137633 | 299339 |
| 1 | Tnfaip8l2 | NM_001014039 | 310663 |
| 1 | Tnfsf4 | NM_053552 | 89814 |
| 1 | Tox2 | NM_199392 | 311615 |
| 1 | Trem2 | NM_001106884 | 301227 |
| 1 | Trh | NM_013046 | 25569 |
| 1 | Trim25 | NM_001009536 | 494338 |
| 1 | Tspo | NM_012515 | 24230 |
| 1 | Ttk | NM_001108172 | 315852 |
| 1 | Tubb6 | NM_001025675 | 307351 |
| 1 | Tyrobp | NM_212525 | 361537 |
| 1 | Uba7 | NM_001106856 | 301000 |
| 1 | Ugt2b34 | NM_001191676 | 305264 |
| 1 | V1rm4 | NM_001008963 | 365190 |
| 1 | Vat1 | NM_001033683 | 287721 |
| 1 | Vav1 | NM_012759 | 25156 |
| 1 | Vim | NM_031140 | 81818 |
| 1 | Vnn1 | NM_001025623 | 29142 |
| 1 | Wap | NM_053751 | 114596 |
| 1 | Znf697 | XM_001064717 | 295310 |
| 2 | Aagab | NM_134398 | 171435 |
| 2 | Aarsd1 | NM_001034109 | 619440 |
| 2 | Abtb2 | NM_134403 | 171440 |
| 2 | Acap1 | NM_001105796 | 287443 |
| 2 | Ano7 | NM_001004071 | 367318 |
| 2 | Asb9 | NM_001191913 | 367785 |
| 2 | C9 | NM_057146 | 117512 |
| 2 | Ccdc69 | NM_001109031 | 497906 |
| 2 | Ccr1l1 | NM_001106872 | 301088 |
| 2 | Ccr8 | XM_001077746 | 301066 |
| 2 | Cd3d | NM_013169 | 25710 |
| 2 | cDNA clone | BI301942 |  |
| 2 | Cfi | NM_024157 | 79126 |
| 2 | Cish | NM_031804 | 83681 |
| 2 | Cldnd2 | NM_001109143 | 499140 |
| **Pattern** | **Symbol** | **Accession** | **Entrez ID** |
| 2 | clones RNOR03327639 | AABR03128582 |  |
| 2 | Crtc3 | XM_001066604 | 365297 |
| 2 | Cst10 | NM_001108961 | 366219 |
| 2 | Ctrb1 | NM_012536 | 24291 |
| 2 | Cyp2s1 | NM_001107495 | 308445 |
| 2 | Cyr61 | NM_031327 | 83476 |
| 2 | Defa | NM_173329 | 286995 |
| 2 | Dok7 | NM_001130062 | 305448 |
| 2 | Drd5 | NM_012768 | 25195 |
| 2 | Ecel1 | NM_021776 | 60417 |
| 2 | Elfn1 | NM_001105913 | 288512 |
| 2 | ENSRNOT00000015993 | XR_006986 |  |
| 2 | ENSRNOT00000023181 | XM_002727495 |  |
| 2 | ENSRNOT00000029966 | XM_001065216 |  |
| 2 | ENSRNOT00000030164 | XM_001078978 |  |
| 2 | ENSRNOT00000034798 | XM_002726273 |  |
| 2 | ENSRNOT00000035914 | XM_001057861 |  |
| 2 | ENSRNOT00000038253 | XM_001058060 |  |
| 2 | ENSRNOT00000041345 | XM_002724546 |  |
| 2 | ENSRNOT00000043066 | XM_001060387 |  |
| 2 | ENSRNOT00000043866 | AY539917 |  |
| 2 | ENSRNOT00000047751 | XM_001067238 |  |
| 2 | ENSRNOT00000051657 | AJ277668 |  |
| 2 | ENSRNOT00000055948 | XR_005454 |  |
| 2 | ENSRNOT00000056186 | XM_001080162 |  |
| 2 | ENSRNOT00000059275 | XM_001059607 |  |
| 2 | ENSRNOT00000059958 | XM_233450 |  |
| 2 | ENSRNOT00000060213 | XM_001056138 |  |
| 2 | ENSRNOT00000061599 |  |  |
| 2 | ENSRNOT00000064915 |  |  |
| 2 | ENSRNOT00000065986 | XM_002728214 |  |
| 2 | ENSRNOT00000066559 | XM_226931 |  |
| 2 | Epha10 | NM_001135709 | 298528 |
| 2 | Etv3 | NM_001106450 | 295297 |
| 2 | Fadd | NM_152937 | 266610 |
| 2 | Fbxl7 | NM_001108545 | 361907 |
| 2 | Fbxo39 | NM_001039018 | 303287 |
| 2 | Fmo13 | XM_001075840 | 289193 |
| 2 | Fmo6 | XM_222818 | 304922 |
| 2 | Fosb | XM_002725540 | 308411 |
| **Pattern** | **Symbol** | **Accession** | **Entrez ID** |
| 2 | Foxe3 | XM_001069443 | 171302 |
| 2 | Galc | NM_001005888 | 314360 |
| 2 | Galp | NM_022633 | 64568 |
| 2 | Gja6 | NM_019308 | 54256 |
| 2 | Gpr142 | XM_001081645 | 501735 |
| 2 | Gpr152 | XM_002725764 | 688884 |
| 2 | Gpr63 | NM_001106640 | 297952 |
| 2 | Gzma | NM_153468 | 266708 |
| 2 | Hist1h4m | XM_001061900 | 291152 |
| 2 | Htr2b | NM_017250 | 29581 |
| 2 | Hydin | XM_226468 | 292017 |
| 2 | Il1f9 | NM_001113790 | 499744 |
| 2 | Iqcf1 | NM_001109361 | 503227 |
| 2 | Itpripl1 | NM_001025043 | 499885 |
| 2 | Kb21 | NM_001008814 | 407761 |
| 2 | Kcnrg | NM_001191687 | 305947 |
| 2 | Krt23 | NM_001008753 | 287678 |
| 2 | Lce1m | NM_001109500 | 688413 |
| 2 | Lct | NM_053841 | 116569 |
| 2 | LOC100361577 | XM_002730109 | 100361577 |
| 2 | LOC100365551 | XM_002727224 | 100365551 |
| 2 | LOC100365687 | XM_002725996 | 100365687 |
| 2 | LOC301289 | NM_001106897 | 301289 |
| 2 | LOC363060 | NM_001014209 | 363060 |
| 2 | LOC680164 | XM_001055979 |  |
| 2 | LOC681303 | XM_002725159 | 681303 |
| 2 | LOC682666 | XM_002728534 | 682666 |
| 2 | LOC685501 | XM_001064056 | 685501 |
| 2 | LOC686396 | XM_227520 | 686396 |
| 2 | LOC687813 | XM_001080233 | 687813 |
| 2 | LOC688420 | XM_001066865 | 688420 |
| 2 | LOC688695 | XM_001067956 | 688695 |
| 2 | LOC688741 | XM_002724948 | 688741 |
| 2 | LOC689927 | NM_001109555 | 689927 |
| 2 | Lrrc25 | XM_573882 | 498605 |
| 2 | Nnmt | NM_001106819 | 300691 |
| 2 | Nyx | NM_001100967 | 302516 |
| 2 | Olr1064 | NM_001001076 | 405966 |
| 2 | Olr1088 | XM_234873 | 299582 |
| 2 | Olr1135 | NM_001000878 | 405177 |
| **Pattern** | **Symbol** | **Accession** | **Entrez ID** |
| 2 | Olr1144 | NM_001000876 | 405174 |
| 2 | Olr1245 | NM_001000809 | 405095 |
| 2 | Olr1264 | NM_001000456 | 300583 |
| 2 | Olr1303 | NM_001000796 | 405080 |
| 2 | Olr132 | NM_001001273 | 293271 |
| 2 | Olr1338 | NM_001000789 | 405073 |
| 2 | Olr1349 | NM_001000484 | 301611 |
| 2 | Olr1436 | NM_001000014 | 287324 |
| 2 | Olr1525 | NM_001000529 | 363710 |
| 2 | Olr1547 | NM_001001107 | 406003 |
| 2 | Olr1558 | NM_001000725 | 404978 |
| 2 | Olr157 | NM_001000169 | 293304 |
| 2 | Olr1606 | NM_001000502 | 305711 |
| 2 | Olr1714 | NM_214456 | 294175 |
| 2 | Olr1736 | NM_001001421 | 405203 |
| 2 | Olr237 | NM_001000210 | 293382 |
| 2 | Olr288 | NM_001000231 | 293590 |
| 2 | Olr56 | NM_001000130 | 293205 |
| 2 | Olr560 | NM_001000325 | 295801 |
| 2 | Olr689 | NM_001000997 | 405357 |
| 2 | Olr704 | NM_001000626 | 404818 |
| 2 | Olr77 | NM_001001287 | 405907 |
| 2 | Olr80 | NM_001001270 | 293223 |
| 2 | Olr862 | NM_001001071 | 405961 |
| 2 | Oprd1 | NM_012617 | 24613 |
| 2 | P4ha3 | NM_198775 | 361612 |
| 2 | Ppp1r3b | NM_138912 | 192280 |
| 2 | Prl8a3 | NM_020079 | 24658 |
| 2 | Prss36 | NM_001011560 | 497040 |
| 2 | Ptchd3 | XM_001081846 | 688333 |
| 2 | Ptgir | NM_001077644 | 292661 |
| 2 | Pth2 | NM_001109144 | 499149 |
| 2 | Ranbp3l | XM_215498 |  |
| 2 | Rcvrn | NM_080901 | 140936 |
| 2 | Rdh8 | NM_001168593 | 690953 |
| 2 | Reg3a | NM_172077 | 171162 |
| 2 | Retnlg | NM_181625 | 288135 |
| 2 | Rexo1 | NM_001012114 | 314630 |
| 2 | RGD1308117 | NM_001134571 | 361066 |
| 2 | RGD1308775 | NM_001107233 | 305476 |
| **Pattern** | **Symbol** | **Accession** | **Entrez ID** |
| 2 | RGD1310251 | NM_001024240 | 291675 |
| 2 | RGD1310376 | NM_001106676 | 298404 |
| 2 | RGD1311870 | XM_001069522 | 298563 |
| 2 | RGD1559508 | XM_577489 | 502056 |
| 2 | RGD1559971 | XM_574786 | 499462 |
| 2 | RGD1563270 | XM_001053319 | 302172 |
| 2 | RGD1563458 | NM_001107349 | 306883 |
| 2 | RGD1563578 | XM_576413 | 501003 |
| 2 | RGD1564240 | XM_002727347 | 290177 |
| 2 | RGD1564496 | XM_574970 | 499650 |
| 2 | RGD1564571 | XM_221808 | 304197 |
| 2 | RGD1564961 | XM_226650 | 294622 |
| 2 | RGD1565953 | XM_224520 |  |
| 2 | RGD1566226 | NM_001134515 | 296149 |
| 2 | Rhox2 | NM_001025746 | 363439 |
| 2 | Scnn1b | NM_012648 | 24767 |
| 2 | Sfrp1 | XM_224987 | 84402 |
| 2 | Similar to hypothetical protein MGC10646 | XM_229903 |  |
| 2 | Similar to olfactory receptor MOR111-1 | XM_222344 |  |
| 2 | Slc12a1 | NM_019134 | 25065 |
| 2 | Slc16a12 | XM_001079987 | 309525 |
| 2 | Slc27a2 | NM_031736 | 65192 |
| 2 | Slc6a12 | NM_017335 | 50676 |
| 2 | Socs3 | NM_053565 | 89829 |
| 2 | Spink5 | NM_001170606 | 361319 |
| 2 | Sucnr1 | NM_001001518 | 408199 |
| 2 | Synpo | NM_021695 | 60324 |
| 2 | Taar5 | NM_001009650 | 294123 |
| 2 | Taar7b | NM_175586 | 294126 |
| 2 | Taar8c | NM_175600 | 319105 |
| 2 | Tas1r1 | NM_053305 | 29407 |
| 2 | TC588511 |  |  |
| 2 | TC590313 | XM_001080179 | 687797 |
| 2 | Tmem104 | NM_001191655 | 303670 |
| 2 | Tmprss6 | NM_001130556 | 315388 |
| 2 | Tpbpa | NM_172073 | 64509 |
| 2 | Tpc1808 | NM_022625 | 64560 |
| 2 | Unc45b | NM_001107028 | 303373 |
| 2 | V1rc37 | NM_001008926 | 494274 |
| 2 | V1rd6 | NM_001008923 | 494272 |
| **Pattern** | **Symbol** | **Accession** | **Entrez ID** |
| 2 | V1rk1 | NM_001008951 | 494296 |
| 2 | Vax1 | NM_022636 | 64571 |
| 2 | Vcsa1 | NM_012684 | 24867 |
| 2 | Vom2r23 | NM_001099649 | 308303 |
| 2 | Vom2r35 | NM_001099473 | 292615 |
| 2 | Vom2r55 | NM_001099495 | 691442 |
| 2 | Zp3r | NM_182815 | 289010 |
