# Supplementary material for: Biphasic Functional Regulation in Hippocampus of Rat with Chronic Cerebral Hypoperfusion Induced by Permanent Occlusion of Bilateral Common Carotid Artery
Source: PLoS One. 2013 Jul 30;8(7):e70093. doi: 10.1371/journal.pone.0070093 (PMC3728362; doi:10.1371/journal.pone.0070093)
Supplement: Table S2 — Full list of GO-terms significantly enriched (FDR<0.01) in Pattern 1 and Pattern 2 in the hippocampus of rats with BCCAO surgery. (DOCX) [file pone.0070093.s005.docx]

Table S2. Full list of GO-terms significantly enriched (FDR<0.01) in Pattern 1 and Pattern 2 in the hippocampus of rats with BCCAO surgery.

| **GO ID** | **GO terms** | **P-value*** | **FDR**** |
| --- | --- | --- | --- |
| **Pattern 1** |  |  |  |
| GO:0006955 | Immune response | 1.58E-29 | 2.71E-26 |
| GO:0006952 | Defense response | 2.23E-26 | 3.85E-23 |
| GO:0006954 | Inflammatory response | 5.66E-21 | 9.76E-18 |
| GO:0002252 | Immune effector process | 1.35E-19 | 2.33E-16 |
| GO:0009611 | Response to wounding | 1.48E-18 | 2.55E-15 |
| GO:0002443 | Leukocyte mediated immunity | 1.13E-15 | 1.91E-12 |
| GO:0002684 | Positive regulation of immune system process | 2.67E-15 | 4.60E-12 |
| GO:0002253 | Activation of immune response | 1.36E-14 | 2.35E-11 |
| GO:0050778 | Positive regulation of immune response | 7.54E-14 | 1.30E-10 |
| GO:0002449 | Lymphocyte mediated immunity | 1.10E-13 | 1.89E-10 |
| GO:0002250 | Adaptive immune response | 4.32E-13 | 7.44E-10 |
| GO:0002460 | Adaptive immune response based on somatic recombination of immune receptors built from immunoglobulin superfamily domains | 4.32E-13 | 7.44E-10 |
| GO:0016064 | Immunoglobulin mediated immune response | 7.33E-13 | 1.26E-09 |
| GO:0045087 | Innate immune response | 1.05E-12 | 1.82E-09 |
| GO:0019724 | B cell mediated immunity | 1.39E-12 | 2.40E-09 |
| GO:0048584 | Positive regulation of response to stimulus | 1.37E-11 | 2.36E-08 |
| GO:0001775 | Cell activation | 2.48E-11 | 4.27E-08 |
| GO:0006959 | Humoral immune response | 5.83E-11 | 1.00E-07 |
| GO:0045321 | Leukocyte activation | 3.88E-10 | 6.69E-07 |
| GO:0006956 | Complement activation | 4.39E-10 | 7.56E-07 |
| GO:0002526 | Acute inflammatory response | 4.60E-10 | 7.93E-07 |
| GO:0002541 | Activation of plasma proteins involved in acute inflammatory response | 6.35E-10 | 1.09E-06 |
| GO:0002455 | Humoral immune response mediated by circulating immunoglobulin | 7.62E-09 | 1.31E-05 |
| GO:0042330 | Taxis | 8.83E-09 | 1.52E-05 |
| GO:0006935 | Chemotaxis | 8.83E-09 | 1.52E-05 |
| GO:0002504 | Antigen processing and presentation of peptide or polysaccharide antigen via MHC class II | 1.25E-08 | 2.15E-05 |
| GO:0006958 | Complement activation, classical pathway | 9.05E-08 | 1.56E-04 |
| GO:0051605 | Protein maturation by peptide bond cleavage | 1.20E-07 | 2.07E-04 |
| GO:0002478 | Antigen processing and presentation of exogenous peptide antigen | 1.37E-07 | 2.35E-04 |
| GO:0002673 | Regulation of acute inflammatory response | 1.38E-07 | 2.38E-04 |
| **GO ID** | **GO terms** | **P-value*** | **FDR**** |
| **Pattern 1** |  |  |  |
| GO:0002495 | Antigen processing and presentation of peptide antigen via MHC class II | 2.72E-07 | 4.69E-04 |
| GO:0019886 | Antigen processing and presentation of exogenous peptide antigen via MHC class II | 2.72E-07 | 4.69E-04 |
| GO:0030593 | Neutrophil chemotaxis | 2.90E-07 | 4.99E-04 |
| GO:0019884 | Antigen processing and presentation of exogenous antigen | 7.72E-07 | 0.001329 |
| GO:0002274 | Myeloid leukocyte activation | 9.71E-07 | 0.001674 |
| GO:0002697 | Regulation of immune effector process | 2.00E-06 | 0.003454 |
| GO:0050900 | Leukocyte migration | 2.68E-06 | 0.00462 |
| GO:0002764 | Immune response-regulating signal transduction | 2.68E-06 | 0.00462 |
| GO:0002768 | Immune response-regulating cell surface receptor signaling pathway | 3.58E-06 | 0.006165 |
| GO:0016485 | Protein processing | 4.55E-06 | 0.007842 |
| **Pattern 2** |  |  |  |
| GO:0007186 | G-protein coupled receptor protein signaling pathway | 9.83E-16 | 1.55E-12 |
| GO:0007600 | Sensory perception | 1.03E-12 | 1.60E-09 |
| GO:0007166 | Cell surface receptor linked signal transduction | 1.84E-12 | 2.86E-09 |
| GO:0007606 | Sensory perception of chemical stimulus | 5.77E-12 | 8.96E-09 |
| GO:0050890 | Cognition | 6.95E-12 | 1.08E-08 |
| GO:0050911 | Detection of chemical stimulus involved in sensory perception of smell | 3.98E-11 | 6.17E-08 |
| GO:0050907 | Detection of chemical stimulus involved in sensory perception | 5.51E-11 | 8.56E-08 |
| GO:0009593 | Detection of chemical stimulus | 8.65E-11 | 1.34E-07 |
| GO:0007608 | Sensory perception of smell | 1.05E-10 | 1.62E-07 |
| GO:0051606 | Detection of stimulus | 1.10E-10 | 1.70E-07 |
| GO:0050906 | Detection of stimulus involved in sensory perception | 1.11E-10 | 1.73E-07 |
| GO:0050877 | Neurological system process | 5.48E-10 | 8.51E-07 |

* P-values were calculated using Fischer’s test.

** FDR corrections were calculated using the Benjamini-Hochberg procedure in DAVID program.
